# Cross Talk Mechanism among EMT, ROS, and Histone Acetylation in Phorbol Ester-Treated Human Breast Cancer MCF-7 Cells

**DOI:** 10.1155/2016/1284372

**Published:** 2016-03-31

**Authors:** Tetsuro Kamiya, Aki Goto, Eri Kurokawa, Hirokazu Hara, Tetsuo Adachi

**Affiliations:** Laboratory of Clinical Pharmaceutics, Gifu Pharmaceutical University, 1-25-4 Daigaku-nishi, Gifu 501-1196, Japan

## Abstract

Epithelial-mesenchymal transition (EMT) plays a pivotal role in the progression of cancer, and some transcription factors including Slug and Snail are known to be involved in EMT processes. It has been well established that the excess production of reactive oxygen species (ROS) and epigenetics such as DNA methylation and histone modifications participate in carcinogenesis; however, the cross talk mechanism among EMT, ROS, and epigenetics remains unclear. In the present study, we demonstrated that the treatment of human breast cancer MCF-7 cells with phorbol ester (TPA), a protein kinase C activator, significantly induced cell proliferation and migration, and these were accompanied by the significant induction of Slug expression. Moreover, the TPA-elicited induction of Slug expression was regulated by histone H3 acetylation and NADPH oxidase (NOX) 2-derived ROS signaling, indicating that ROS and histone acetylation are involved in TPA-elicited EMT processes. We herein determined the cross talk mechanism among EMT, ROS, and histone acetylation, and our results provide an insight into the progression of cancer metastasis.

## 1. Introduction

Accumulated evidence shows that the excess generation of reactive oxygen species (ROS) elicits oxidative stress in cells and tissues and leads to various diseases, such as cancer, atherosclerosis, and type 2 diabetes [1–3]. A recent study demonstrated that epithelial-mesenchymal transition (EMT) plays a pivotal role in cancer metastasis [4], including breast cancer, which is the most common malignancy in Japanese women. The expression of Slug and Snail, which are key transcription factors in EMT processes, was previously found to be increased in cancer tissues and has been closely associated with EMT phenomena [5–7]. EMT is characterized by the loss of epithelial-like properties including the tight-junction proteins, E-cadherin and N-cadherin [8–10], and the acquisition of mesenchymal properties such as the extracellular matrix protein fibronectin-1 [11, 12]. These processes increase aggressiveness and enhance the metastatic spread of breast cancer [13]; therefore, identifying key molecules in EMT and elucidating the mechanisms underlying it may ultimately result in the suppression of breast cancer malignancy.

Epigenetics, such as DNA methylation and histone modifications, are typically referred to as mitotically heritable changes in gene expression that do not involve any changes in DNA sequences [14]. DNA methyltransferases (DNMTs) 1, 3A, and 3B are known to play critical roles in DNA methylation processes by using S-adenosyl methionine as a methyl donor [15]. Previous studies demonstrated that global DNA hypomethylation and regional hypermethylation are related to the initiation and progression of tumorigenesis [16–18]. Hypermethylation of the* p53* promoter region, which decreases its expression, has been suggested to lead to tumor progression [19–21]. On the other hand, histone modifications including acetylation and methylation at arginine or lysine residues are also associated with gene expression and silencing [22–24]. Among histone modifications, the histone acetylation status is regulated by histone deacetylase (HDAC) and/or histone acetyltransferase (HAT) [25–27]. Recent studies showed that the expression of E-cadherin was regulated by its DNA hypermethylation in hepatocellular carcinoma (HCC) tissues [28]; however, the role of histone modifications in EMT processes, especially in the regulation of the expression of transcriptional factors, remains unclear.

In the present study, we examined the induction of Slug expression in phorbol ester- (TPA-) treated human breast cancer MCF-7 cells. The results obtained indicated that the TPA-elicited induction of Slug expression is associated with histone H3 acetylation within its promoter region, and these processes are due to the excess production of NADPH oxidase- (NOX-) derived ROS. Taken together, these results contribute to a deeper understanding of the significant role of ROS in EMT processes and epigenetic gene regulation and may lead to the development of novel epigenetic therapies for breast cancer.

## 2. Materials and Methods

### 2.1. Materials

TPA and HRP-conjugated goat anti-rabbit (A6154) and anti-mouse (A4416) IgG were purchased from Sigma-Aldrich Co. (St. Louis, MO). A PKC inhibitor (GF109203X) and actinomycin D (ActD) were purchased from Wako Pure Chemical Industries, Ltd. (Osaka, Japan). Cyclopentylidene-(4-(4′-chlorophenyl)thiazol-2-yl)hydrazone (CPTH2) was purchased from Calbiochem (San Diego, CA). 5-(and-6)-Carboxy-2′,7′-dichlorodihydrofluorescein diacetate (carboxy-H_2_DCFHDA) and dihydroethidium (DHE) were purchased from Molecular Probes (Eugene, OR). Diphenyleneiodonium (DPI) and garcinol (Gar) were purchased from Enzo Life Sciences Inc. (Farmingdale, NY). Trichostatin A (TSA) was purchased from Cayman Chemical (Ann Arbor, MI). An anti-phospho-PKC (pan) (*β*II Ser660) rabbit polyclonal antibody (#9371) and normal rabbit IgG (#2729) were purchased from Cell Signaling Technology (Danvers, MA). Anti-actin mouse monoclonal antibody (MAB1501) and anti-acetyl-histone H3 (#06-599) and H4 (#06-598) rabbit polyclonal antibodies were purchased from Millipore Co. (Billerica, MA).

### 2.2. Cell Culture

MCF-7 cells were cultured in Dulbecco's modified Eagle's medium (DMEM) supplemented with 10% fetal calf serum (FCS), 100 units/mL penicillin, and 100 *μ*g/mL streptomycin and maintained at 37°C in a humidified 5% CO_2_ incubator. Cells were grown to confluence on a 96-well plate (seeded at 1 × 10^4^ cells/well or 2 × 10^4^ cells/well), 4-well plate (seeded at 5 × 10^4^ cells/well), 6 cm culture dish (seeded at 3 × 10^5^ cells/dish), or 10 cm culture dish (seeded at 1 × 10^6^ cells/dish) and treated with the reagents described below.

### 2.3. Wound-Healing Assay

After cells became confluent, a wound field was prepared using a pipette tip. Culture medium was then replaced with fresh medium containing 20 *μ*M DPI and incubated for 1 h. The wound field 24 or 72 h after the TPA treatment (1 nM) was examined under a microscope.

### 2.4. Cell Proliferation Assay

MCF-7 cells were seeded on 96-well plate at 1 × 10^4^ cells/well and grown for 12 h. After that, the cells were treated with or without 1 nM TPA for 12 h following incubation for 2 h with Cell Counting Kit-8 (CCK-8) assay reagent (Dojindo, Japan). The colorimetric intensity at 450 nm of each well was measured using the iMark*™* microplate reader (BioRad Lab, Hercules, CA).

### 2.5. PCR Analysis

After MCF-7 cells had been treated, they were lysed in 1 mL TRIzol® reagent (Invitrogen, Carlsbad, CA). The cDNA preparation and RT-PCR were performed using the methods described in our previous study [29]. The primer sequences used in the present study are shown in Table 1. These PCR products were loaded onto a 2% (w/v) agarose gel for electrophoresis, and a densitometric analysis of PCR products was performed with Multi Gauge version 3.0 (Fuji Film, Tokyo, Japan).

### 2.6. Western Blotting

Whole cell protein from MCF-7 cells was prepared as described in our previous study with minor modifications. Briefly, cells were lysed in lysis buffer (20 mM Tris-HCl, pH 7.4, containing 1% Triton X-100, 1 mM EDTA, 1 mM EGTA, 10 mM NAF, 1 mM Na_3_VO_4_, 20 mM *β*-glycerophosphate, 1 mM DTT, and 1 mM PMSF), followed by sonication using the ultrasonic homogenizer Vivracell VC100 (Sonic & Materials, Danbury, CT). Cytosolic and membrane fractions were isolated as described previously [30]. Whole cell, cytosolic, or membrane protein concentrations were measured by a BCA protein assay. Twenty micrograms of protein was boiled with SDS buffer (62.5 mM Tris-HCl, pH 6.8, containing 5% 2-mercaptoethanol, 2% SDS, 10% glycerol, and 0.01% bromophenol blue (BPB)) for 5 min. Core histones were isolated as described in our previous report [29] and boiled with SDS buffer for 5 min. Whole cell, cytosolic, or membrane protein (20 *μ*g) or isolated histones from approximately 5 × 10^4^ cells were separated by SDS-PAGE on a 12 or 15% (w/v) polyacrylamide gel, followed by their transferal electrophoretically onto PVDF membranes. The membranes were then incubated with anti-phospho-PKC (pan) (*β*II Ser660) rabbit polyclonal antibody (#9371, 1 : 1,000), anti-acetyl-histone H3 rabbit polyclonal antibody (#06-599, 1 : 1,000), anti-acetyl-histone H4 rabbit polyclonal antibody (#06-598, 1 : 1,000), or anti-actin mouse monoclonal antibody (MAB1501, 1 : 3,000). The blots were incubated with HRP-conjugated goat anti-rabbit (A6154) or anti-mouse (A4416) IgG (1 : 5,000). Bands were detected using ImmunoStar®LD and imaged using LAS-3000 UV mini (Fuji Film).

### 2.7. ChIP Analysis

ChIP assays were performed as described in our previous report with minor modifications [29]. The sheared genomic DNA was immunoprecipitated with normal rabbit IgG (#2729), anti-acetyl-histone-H3 (#06-599) or anti-acetyl-histone-H4 rabbit polyclonal antibody (#06-598) for overnight followed by incubation with Dynabeads protein G (Invitrogen) for 2 h. The abundance of Slug or Snail promoter regions in ChIP precipitates was quantified using a PCR analysis. The primer sequences used in the ChIP assay were as follows: Slug: sense 5′-GAG GTT CCT CTC TTG AAA ATA CT-3′, antisense 5′-GCA AGA AAG ATC CAA TCA CA-3′; Snail: sense 5′-CGC TCC GTA AAC ACT GGA TAA-3′, antisense 5′-GAA GCG AGG AAA GGG ACA C-3′. After amplification, these PCR products were loaded onto a 1.2% (w/v) agarose gel for electrophoresis and visualized using FLA5100, and a densitometric analysis of PCR products was performed with Multi Gauge version 3.0.

### 2.8. Measurement of HDAC Activities

HDAC activities were measured in TPA-treated MCF-7 cells using the HDAC Cell-Based Activity Assay Kit (Cayman Chemical Company, Ann Arbor, MI) according to the manufacturer's protocol. Briefly, after MCF-7 cells had been treated with 1 nM TPA for 1 h, they were incubated at 37°C for 2 h in the presence of the HDAC substrate. Lysis/developer solution was added to the cells, followed by incubation at 37°C for 15 min. The fluorescent intensity (excitation 365 nm, emission 410–460 nm) of each well was measured using the GloMax®-Multi Detection System (Promega, Madison, WI).

### 2.9. Determination of Intracellular ROS Accumulation

After MCF-7 cells had been treated, the cells were incubated with PBS containing 5% paraformaldehyde and 10 *μ*M carboxy-H_2_DCFHDA or 10 *μ*M DHE for 20 min at 37°C in a humidified 5% CO_2_ incubator. The cells were visualized under an HS All in One fluorescence microscope BZ-9000 (Keyence, Osaka, Japan).

### 2.10. Statistical Analysis

Data are expressed as the means ± SD of three independent experiments. Statistical evaluations of the data obtained were performed using an ANOVA followed by Bonferroni* post hoc* tests or Student's *t*-test for Figures 3(b) and 3(d). A *p* value less than 0.05 was considered significant.

## 3. Results

### 3.1. Treatment with TPA Induces EMT Processes in Human Breast Cancer MCF-7 Cells

**Figure 1:**
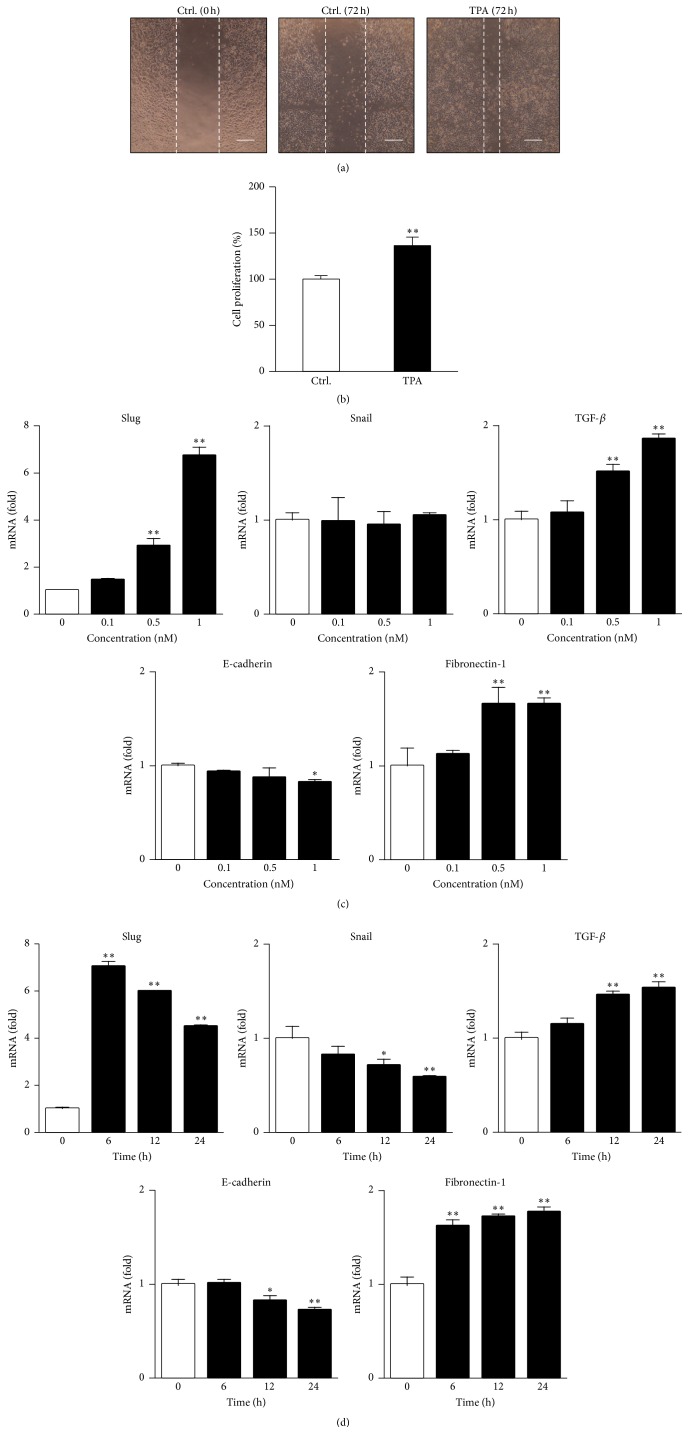
TPA treatment induces EMT processes in human breast cancer MCF-7 cells. (a) MCF-7 cell migration into the wound field was monitored by the method described in Section 2. The scale bars show 200 *μ*m. (b) MCF-7 cells were treated with 1 nM TPA for 12 h. After that, the cell proliferation was evaluated by CCK-8 assay (^*∗∗*^ *p* < 0.01 versus vehicle). MCF-7 cells were treated with the indicated concentrations of TPA for 24 h (c) or 1 nM TPA for the indicated times (d). RT-PCR was then carried out. RT-PCR data were normalized using *β*-actin levels (^*∗*^ *p* < 0.05, ^*∗∗*^ *p* < 0.01 versus vehicle (c) or 0 h (d)).

PKC plays a pivotal role in many physiological processes including cancer metastasis [31, 32], which is associated with cell proliferation and migration, the induction of EMT-related genes, and reductions in tight-junction molecules. Therefore, we investigated MCF-7 cell migration after the TPA treatment using the wound-healing assay. MCF-7 cell migration was not observed under serum-free conditions; however, the treatment with TPA significantly induced its migration (Figure 1(a)). Moreover, it was determined that treatment with TPA significantly induced MCF-7 cells proliferation (Figure 1(b)), suggesting that TPA functions as a potent cell migration and proliferation inducer in this model. Slug and Snail, major EMT-related transcription factors, are known to induce fibronectin-1 and decrease E-cadherin expression during the progression of tumor metastasis. As shown in Figures 1(c) and 1(d), the treatment with TPA significantly induced the expression of Slug, whereas it did not induce that of Snail in MCF-7 cells. Moreover, we demonstrated the induction of transforming growth factor-*β* (TGF-*β*) and fibronectin-1 and reductions in E-cadherin in TPA-treated MCF-7 cells, suggesting that TPA induces EMT processes through Slug-related signaling in MCF-7 cells.

### 3.2. Involvement of PKC in TPA-Induced EMT Processes in MCF-7 Cells

**Figure 2:**
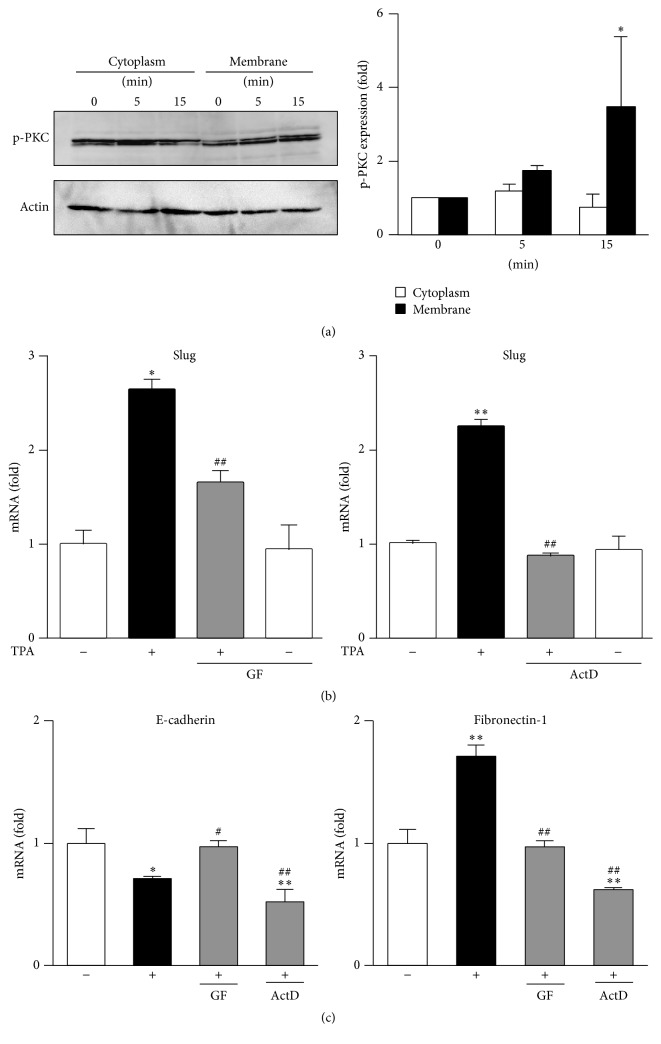
Involvement of PKC in TPA-induced EMT processes in MCF-7 cells. (a) MCF-7 cells were treated with 1 nM TPA for the indicated times. The membrane translocation of phosphorylated PKC was then determined by Western blotting. Values are the mean of fold changes from vehicle-treated cells (*n* = 3, ^*∗*^ *p* < 0.05). The cells were pretreated with 5 *μ*M GF109203X (GF) or 10 *μ*g/mL actinomycin D (ActD) for 1 h and were then treated with 1 nM TPA for 6 h (b) or 24 h (c). RT-PCR was carried out. RT-PCR data were normalized using *β*-actin levels (^*∗*^ *p* < 0.05, ^*∗∗*^ *p* < 0.01 versus vehicle, ^#^ *p* < 0.05, ^##^ *p* < 0.01 versus TPA-treated cells).

In order to determine the involvement of PKC in TPA-induced EMT processes, we demonstrated the membrane translocation of phospho-PKC in TPA-treated MCF-7 cells. As shown in Figure 2(a), the treatment with TPA induced the membrane translocation of phospho-PKC more rapidly than in control cells, indicating that TPA activated PKC signaling, which is consistent with previous findings. We then investigated the inhibitory effects of GF109203X, an inhibitor of PKC, on TPA-elicited gene alterations. The pretreatment with GF109203X suppressed the induction of TPA-elicited Slug and fibronectin-1 and reductions in E-cadherin (Figure 2(b)), suggesting that PKC signaling is involved in TPA-elicited EMT processes. Moreover, the TPA-elicited induction of Slug expression was completely suppressed by the pretreatment with actinomycin D (ActD), an inhibitor of transcription, indicating that its induction was regulated at the transcription level.

### 3.3. TPA-Elicited Induction of Slug Expression Was Regulated by Histone H3 Acetylation within Its Promoter Region

**Figure 3:**
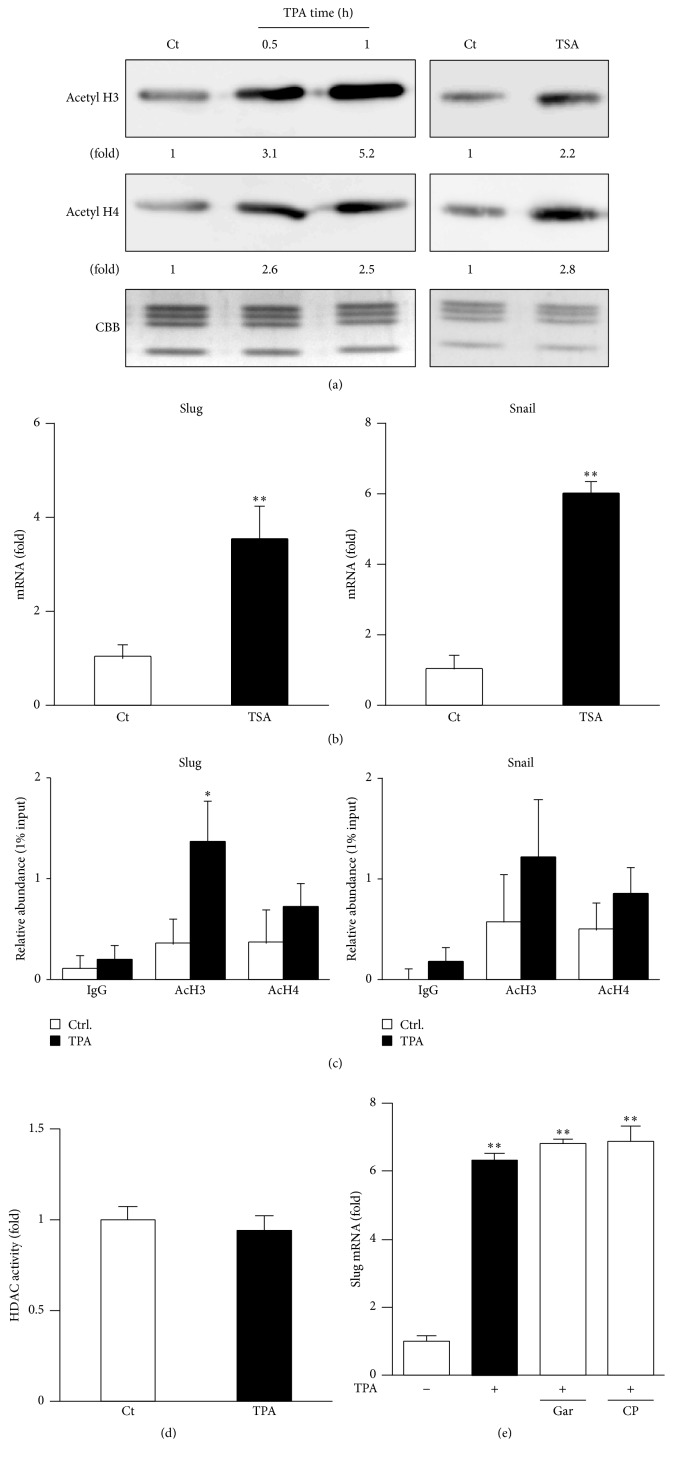
TPA-elicited Slug induction is regulated by histone H3 acetylation within its promoter region. (a) MCF-7 cells were treated with 1 nM TPA or 1 *μ*M of TSA for the indicated times (for TPA) or 1 h (for TSA). Acetylated histones H3 and H4 were then determined by Western blotting. Values are the mean of fold changes from vehicle-treated cells (*n* = 3). (b) Cells were treated with 1 *μ*M TSA for 6 h, followed by RT-PCR. RT-PCR data were normalized using *β*-actin levels (^*∗∗*^ *p* < 0.01 versus vehicle). (c) Cells were treated with 1 nM TPA for 1 h. A ChIP assay was then performed. Relative binding to the promoter region is expressed as the percentage amount over input (%) (^*∗*^ *p* < 0.05 versus vehicle). (d) After cells had been treated with 1 nM TPA for 1 h, HDAC activities were measured. (e) Cells were pretreated with 30 *μ*M garcinol (Gar) or 50 *μ*M CPTH2 (CP) for 30 min and then treated with 1 nM TPA for 6 h, followed by RT-PCR. RT-PCR data were normalized using *β*-actin levels (^*∗∗*^ *p* < 0.01 versus vehicle).

We previously reported that TPA induced superoxide dismutase 3 (SOD3) expression through epigenetics such as histone acetylation in human leukemic THP-1 cells [29]; therefore, TPA-elicited Slug induction may also be regulated by epigenetics. As expected, the TPA treatment induced histones H3 and H4 acetylation in a time-dependent manner (Figure 3(a)), and this induction was stronger than that elicited by TSA. Moreover, the treatment with TSA significantly induced the expression of Slug (Figure 3(b)), and our ChIP assay determined the significant enrichment of acetylated histone H3 within the proximal promoter region of* Slug* (Figure 3(c)). However, the treatment with TSA, but not TPA, significantly induced Snail expression. These results suggest that the treatment with TPA selectively induced histone acetylation within the* Slug* promoter region in MCF-7 cells. It has been well established that HDAC and HAT play critical roles in histone acetylation [25–27]; however, the treatment with TPA did not decrease HDAC activities in our model (Figure 3(d)). Furthermore, HAT inhibitors such as Gar and CPTH2 did not suppress the TPA-elicited induction of Slug expression (Figure 3(e)), indicating that TPA induced its expression in a HDAC- and HAT-independent manner.

### 3.4. Involvement of Intracellular ROS in TPA-Elicited EMT Processes in MCF-7 Cells

**Figure 4:**
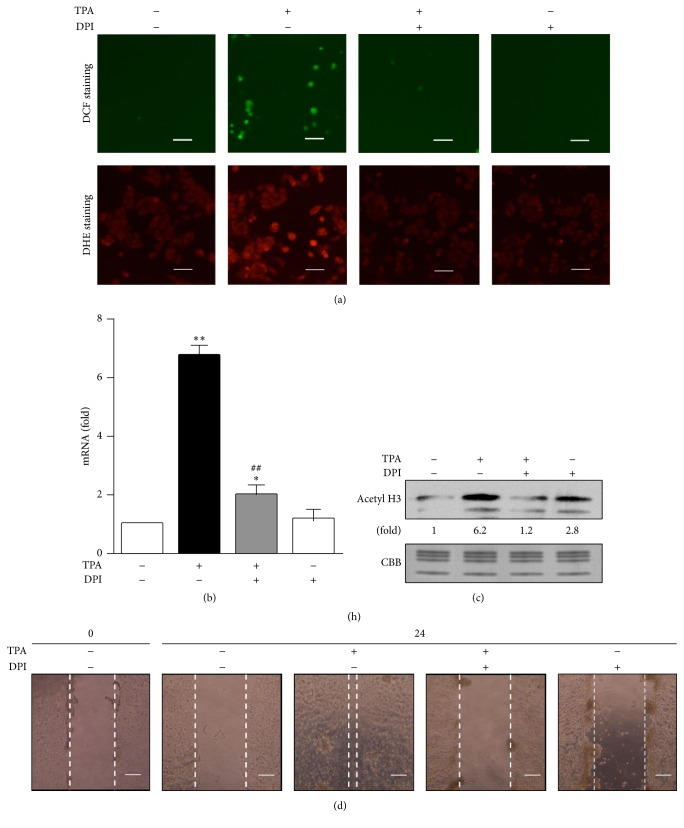
Involvement of intracellular ROS in TPA-elicited EMT processes in MCF-7 cells. MCF-7 cells were pretreated with 20 *μ*M DPI for 1 h and were then treated with 1 nM TPA for 30 min (a), 1 h (b), 6 h (c), or 24 h (d). Intracellular ROS accumulation (a), Slug mRNA expression (b), acetylated histone H3 levels (c), and cell migration (d) were determined. Scale bars show 200 *μ*m. Values (b) are the means of fold changes from vehicle-treated cells (*n* = 3). RT-PCR data were normalized using *β*-actin levels (^*∗*^ *p* < 0.05, ^*∗∗*^ *p* < 0.01 versus vehicle, ^##^ *p* < 0.01 versus TPA-treated cells).

NOX2, the expression of which is the strongest in monocytes/macrophages, plays an essential role in innate host defenses and is now known as a signaling molecule [33, 34]. We previously reported that NOX2-derived ROS after a TPA treatment functioned as key signal molecules in human leukemic U937 cells [30] and THP-1 cells [35]. In the present study, intracellular ROS generation was enhanced by the treatment with TPA but was suppressed by the pretreatment with DPI, an inhibitor of NOX2 (Figure 4(a)). Moreover, in the presence of DPI, the TPA-elicited induction of Slug was significantly blocked (Figure 4(b)), and this was accompanied by the inhibition of histone H3 acetylation (Figure 4(c)), indicating that NOX2-derived ROS participate in the TPA-elicited induction of Slug in MCF-7 cells. We then investigated the inhibitory effects of DPI on TPA-elicited MCF-7 cell migration. As shown in Figure 4(d), the pretreatment with DPI markedly suppressed TPA-elicited MCF-7 cell migration, suggesting that NOX2-derived ROS are involved in cell migration as well as the induction of Slug expression.

## 4. Discussion

EMT, which is associated with the loss of epithelial-like properties and the acquisition of mesenchymal properties, is considered to be involved in tumor metastasis and tissue fibrosis [36, 37]. We herein demonstrated that a treatment with TPA, an activator of PKC, induced breast cancer EMT processes through the significant induction of Slug, but not Snail expression. PKCs *α*, *ε*, *η*, *ζ*, and *δ* are known to be significantly involved in cell proliferation, migration, and invasion, particularly in breast cancer [31, 32, 38–42]. Our results showed that PKC signaling plays an important role in the TPA-elicited induction of Slug expression (Figure 2) and is consistent with previous findings suggesting the critical role of PKC signaling in tumor initiation and progression. On the other hand, we also found the significant induction of TGF-*β* (Figure 1(c)). Elevated plasma TGF-*β*1 levels in breast and prostate cancer patients are considered to correlate with poor outcomes [43–45]. TGF-*β*-elicited EMT processes are essential for normal embryonic development but are considered to contribute to tumor cell invasion and metastasis. Therefore, we speculated that elevated TGF-*β* levels might be involved in TPA-elicited EMT processes in MCF-7 cells.

The excess production of ROS is known to contribute to the progression of atherosclerosis, asthma, and cancer [1–3]. Moreover, ROS play a critical role in the induction of Snail expression in HCC tissues and have been closely associated with reductions in E-cadherin [28]. Therefore, a clearer understanding of the role of ROS and regulation of redox homeostasis may lead to the development of novel cancer therapies. In the present study, we determined the involvement of NOX-derived ROS in the TPA-elicited induction of Slug expression, which was closely associated with histone H3 acetylation within its promoter region (Figure 3). These results provide direct evidence for excessively produced ROS regulating the expression of various genes through chromatin remodeling. On the other hand, our results showed that TPA did not induce Snail expression (Figure 1). Nevertheless, the treatment with TSA, an inhibitor of HDAC, significantly induced Slug and Snail expression (Figure 3). These results suggest that TPA selectively induces the expression of Slug through histone H3 acetylation. We previously reported that a treatment with TPA activated HAT including p300 and GCN5, which contribute to the TPA-elicited expression of SOD3 in human leukemic THP-1 cells [29], suggesting that TPA-elicited Slug expression might be associated with the activation of HAT. However, we were unable to determine the involvement of HAT in its induction (Figure 3(e)). Taken together, these results showed that the treatment with TPA did not decrease HDAC activities (Figure 3(d)). A recent study reported that reductions in the levels of Sirtuin 1 (SIRT1), a highly conserved NAD-dependent deacetylase, in breast cancer and kidney tubular epithelial cells promoted tumor metastasis and kidney fibrosis, respectively [46, 47]. Furthermore, SIRT1 deacetylates and suppresses Smad4, a key molecule in TGF-*β* signaling, which lowers the expression of target genes [47]. Therefore, it raises the possibility that inhibition of SIRT families might regulate TPA-elicited histone H3 acetylation and Slug induction in MCF-7 cells; however, some additional experiments are needed in order to elucidate the exact mechanisms governing TPA-elicited EMT processes.

In the present study, we identified a critical role for ROS in the histone H3 acetylation within the Slug promoter in MCF-7 cells. Taken together with previous findings, our results provide the informative evidence for NOX-derived ROS inducing epigenetic modifications. It remains unknown how ROS selectively regulate histone H3 acetylation within the Slug promoter region; however, a clearer understanding of the role of ROS may lead to the development of novel epigenetic therapies for breast cancer.

## Figures and Tables

**Table 1:** Primer sequences used in RT-PCR.

Gene	Sequences
Slug	S: 5′-AGCCAAACTACAGCGAACTG-3′ AS: 5′-GGTCTGAAAGCTTGGACTGT-3′
Snail	S: 5′-CCAATCGGAAGCCTAACTAC-3′ AS: 5′-CTCCAAGGAAGAGACTGAAG-3′
TGF-*β*	S: 5′-ATCGACATGGAGCTGGTGAA-3′ AS: 5′-GTTCAGGTACCGCTTCTCGG-3′
E-cadherin	S: 5′-AGAATGACAACAAGCCCGAAT-3′ AS: 5′-CGGCATTGTAGGTGTTCACA-3′
Fibronectin-1	S: 5′-CCAACCTACGGATGACTCGT-3′ AS: 5′-GCTCATCATCTGGCCATTTT-3′
*β*-actin	S: 5′-CAAGAGATGGCCACGGCTGCT-3′ AS: 5′-TCCTTCTGCATCCTGTCGGCA-3′
